# Neurasthenia in Relation to the Abdominal Diseases of Women*Read before the Johnson County Medical Society, March 12, 1903. Published by request of the Society.

**Published:** 1903-04

**Authors:** Henry K. Leake

**Affiliations:** Dallas, Texas


### TEXAS MEDICAL JOURNAL.

ESTABLISHED JULY, 1885.

PUBLISHED MONTHLY.—SUBSCRIPTION $1.00 A YEAR.

VOL. XVIII. AUSTIN, APRIL, 1903. No. 10.

### Original Contributions.

For Texas Medical Journal.

### Neurasthenia in Relation to the Abdominal Diseases of Women.*

BY HENRY E. LEAKE, M. D., DALLAS, TEXAS.

Allow me to thank you for the kind invitation you gave me to address you upon this occasion, an invitation that I hope you will not discover—from the crude and disjointed character of my remarks—to be unmerited, for the time at my disposal for preparation was brief and other duties pressing. Such a paper, neverless, may have merit from the fact that it may show the superficial side of one's information when not based upon extended thought and research, thus comfortably assuring us of our intellectual equality in ordinary circumstances, besides affording ample scope for discussion and criticism.

I have not come before you with a new operation, nor with a series of old ones recently skillfully performed. I have chosen rather to awaken interest in a familiar topic, and one that daily confronts the general practitioner as well as the specialist. I refer to the so-called neurasthenia in its relation to abdominal diseases of women, although to discuss the subject properly it is necessary to consider the disease more or less in its general aspects. I could hardly be expected to attempt an exhaustive review of the subject, neurasthenia. The specialist, in the treatment of female disease, has neither the time nor the opportunity to go into such details; the

*Read before the Johnson County Medical Society, March 12, 1903. Published by request of the Society.

intricate pathology of neurasthenia in its broadest significance must be left to the neurologist and to the pathologist, although here, as elsewhere in many practical matters, the surgeon and the clinician may suggest to the former the clue for their studies, or even force them to stand upon ground that they might be disinclined to occupy. Neurasthenia is no exception to this latter statement, for the various notions concerning the condition were crystallized into a definite and practical conception by Beard, 1868, who, if I remember aright, determined the modern idea upon a basis of clinical observation and experience, leaving its complex pathology to be more fully elaborated by suggesting the later researches of the pathologist who will reinforce the specialist in nervous diseases. Indeed, the very name "neurasthenia" was given to medical nomenclature by Beard and it yet stands as the best verbal exponent of a condition that affects a large percentage of the community, and expresses itself in varying circumstances and in varying degrees in all the walks of life. Men and women are both subject to this disorder, as are also the rich and the poor. From my own experience I should judge that, contrary to the views of Savill, and I believe Beard himself, women are equally affected with men, if not to a greater extent, although here again my observatoins in this direction have been oftener among women than men. Savill points out the error in the statement that the rich are chiefly affected by this disorder, for he has found that the poor, both numerically and in the same degree, are likewise prone to this distressng affection.

I shall not enter upon a discussion of the strenuous conditions of our American life insisted upon by Beard and others as the cause of this disorder in men and in passing shall only remark that even some of the condition arising out of the social and commercial life of men may operate rarely in the case of women to develop and intensify the neurasthenic condition. The ordinary causes in women are sufficiently numerous, of themselves, to attract your attention, but it appears necessary, for a proper apprehension and understanding of these causes, for the practitioner to have at least a theoretical idea of the basic pathology underlying them, in order that his therapeutics may be as nearly rational, and as practical as possible. I believe that not any author can at the present time give us an absolutely definite idea of the pathology of neurasthenia. So far as I know, all published statements are involved in much obscurity. I know of no pathology that is based upon postmortem examinations, hence it is that our pathological ideas of the disease, no matter how skillfully arranged, or rationally conceived, must be considered little short of speculation, and this speculation has hardly advanced beyond classing the disease as functional, when its persistence and its rebellious character to treatment would seem to prove its organic nature, at least in the advanced stages, so that we deduce the practical observation that this disease should be recognized early, if its ravages are to be forestalled. In our examinations, therefore, we are thoughtful to recognize early the clinical picture of the disease, and in so doing, we are not seldom fortunate in its treatment.

But in order to support some contentions that I shall make later on, it is necessary to consider somewhat the pathology of neurasthenia, or rather the manifestations of its pathology, that is unknown. Neurasthenia is essentially a nervous disease; it must, therefore, be studied as a departure from the normal anatomy and physiology of the nervous system, particularly that of the nervous centres. Moreover, it has been studied by contrast and comparison with other nervous diseases, and chiefly, as was pointed out by Savill, by a method of analogical reasoning, or, if you please, demonstration. So far, this will involve us in much theoretical speculation, but this speculation is not based upon any hypothesis, as is often the case in medicine; it is theory, nevertheless, but is formed not as "the clay model of hypothesis," but of its own ability, it may stand alone upon rigid observation and experience.

The nearest approach to a satisfactory theoretical explanation of this observation and experience, has been attempted recently by Professor Clifford Allbert, of Cambridge University, England. I am forced to quote him at length. He says:

"Diagrammatically, they might conceive of the nervous system as a vertical pile of centres, the lowermost of which presided over the earliest of the functions, each higher center in its turn being occupied with functions later and later in the order of development. Not only so, but each later centre was not only attached directly to its predecessor, but was co-ordinated also with its predecessors, so that the pile became a system of mutual interdependence. Thus, every later system modified, or in other words, controlled or inhibited, not only the one immediately below it, but also each and all the earlier centres, certain cardinal inhibitions being provided by special short circuits. Thus, for example, sphincters became inhibited by higher motor centres, and these, in their turn, by centres of still higher levels. But a nervous centre was not a mere change-house; it was also an accumulator of energy, whereby it was prepared not only for mean demand, but also for extremes of effort or rest. It was not only a fly-wheel which did not enlarge as the output of its engine increased, but rather like a bank which, as the output of the body increased, became itself enriched for direct expenditure, mutual reinforcement and inhibition. In capacity of enrichment, by way of expenditure, individuals differed widely. In some an excessive run might strain the central resources so far that even the needs of ordinary functions were met with difficulty. In one man a certain measure of work not only achieved the immediate end, but also enriched his reserve of energy; in another, if it achieved the immediate end, it fell short of addition to the reserve; in another the end was achieved at the cost of depleting the reserve; in the fourth, even the immediate end was not attained, and the reserve was not only exhausted, but its elasticity was so impaired that, for some time to come, it was unable to keep up with current work. The neurasthenic had never much reserve for time of stress; he needed inordinate time for repair, and was apt to be exhausted beyond the possibility of full repair. In this lowering of nervous potential, not only did output soon run down, but inhibition was slackened so that energy was prematurely exhausted in what they called irritable weakness."

Listen now to the definition of Savill. "I regard neurasthenia as an irritable weakness of the entire nervous system, characterized (when the brain is chiefly affected) by hypersensitiveness of the sensorium, by headache, inaptitude for mental work, disturbed sleep and irritability of temper; and (when the spinal cord is chiefly affected) by general weakness, restlessness, nervousness and vague pains, and usually accompanied (in both forms) by various phenomena referable to the vaso-motor and sympathetic systems."

From all this it appears that neurasthenia embodies a group of symptoms, caused by some definite pathological entity, and whether or not we believe this pathological entity may be developed by some disease within the abdomen, certainly it may exist without any such relationship, in my experience, a fact oftener than other overlooked by the average practitioner. To recognize this group of symptoms that in close alliance would constitute the disease known as neurasthenia, of course we must rely either upon an intuitive or the differential diagnosis. It is not within the scope of this paper to discuss this question fully, but I may remind you that neurasthenia is often simulated by hysteria, hypochondriasis, and by insanity, with which, it is said, it occasionally may be associated, or, as regards the latter, into which it may be transformed. I may refer also, as bearing upon the intent of my paper, to some conditions for which neurasthenia may be mistaken, and in some of which operations have been advised not only, but really undertaken. These conditions are sometimes the result of mere nervous or physical weakness, or both combined, such as may occur in cases of chronic dyspepsia, although in this case neurasthenia must be carefully differentiated, for chronic indigestion is one of its common phenomena and, as is claimed, erroneously it is said, by some authors to be a cause of this disease. The nervous weakness, also seen in many cases of high arterial tension, may be taken for neurasthenia; and even the nervous symptoms caused by the syphilitic poison has sometimes invited, and secured, a diagnosis of neurasthenia with the most disastrous results.

Examples of these kinds I have seen in my practice. I have been impressed particularly by a case, wherein a diagnosis of neurasthenia had been made, but where the symptoms were all caused by constitutional syphilis, unsuspected by competent physicians, who sent her to my hospital for treatment. She seemed to present all the phases of marked neurasthenia that I feared was passing rapidly into insanity. After one week's close study of the case, during which time I was sorely perplexed in my diagnosis, I elicited a remote, but definite clue to her real conditions. Years before she had been infected through her child in utero, and other than the symptoms she now presented, no sign of constitutional syphilis was apparent. Under specific treatment her weight increased from 120 to 170 pounds, and all the symptoms vanished as though by the wand of a magician. Rescued from a life of wretched despair, she has become an animated and contented woman and her recovery has been without relapse. This woman was sent to me as suffering from some pelvic disease that must be eliminated by operative treatment. After examination no local treatment whatever was instituted, for her pelvic organs then, as now, were in an absolutely normal condition.

The remote effects of influenza are said by Allbut and others, to be mistaken for genuine neurasthenia; such cases will sometimes drift into the hands of the specialist in female diseases, to be operated upon for supposed ovarian or uterine disease; and patients with cerebral disease, such a abscess, migraine and some forms of spinal disease will afford a reasonable excuse for an erroneous diagnosis of neurasthenia.

Doubtless these examples, wherein local and general diseases have been overlooked, the diagnosis of neurasthenia made and, perhaps, the patients doomed to unwarrantable operative traumatism, will be added to from your own experience, and that of many others in the profession.

But this error frequently concerns the gynecologist and the specialist in abdominal diseases. I will not hazard a determination of the question, whether or not real neurasthenia is ever caused by abnormal disturbances within the abdomen, including the pelvis, although I may express a doubt in this direction. Certainly the authorities have drawn a marked distinction between the nervous and physical weakness attending many of these conditions, and the disease that they dignify by the term neurasthenia—a disease with special characteristics of its own. They would not deny that the two conditions often are associated, but not as cause and effect, however much the one might seem to aggravate the other, although in the event of an operation designed to eliminate this aggravation, it is discovered afterwards that the nervous element in the case was intensified seriously, if not hopelessly. Clifford Allbutt is of this opinion, that, he says, has been growing upon him steadily in late years, and from my limited experience I can support him heartily in this contention. At this point, I cannot refrain from claiming that the nerve storms, known as "pelvic reflexes," in women, frequently are confounded with this disease of neurasthenia, to remedy which patients have been subjected unwisely to operation. I will go further and cast doubt upon the whole theory of "pelvic reflexes" in disease* (Lasegue) as having no predominant importance, albeit probably a nervous and physical breakdown may accompany, or even be caused by, disease within abdominal cavity, but if there be nervous reflexes, pure and simple from abdominal, and particularly from pelvic disease, then neurasthenia may be regarded, also, as a condition of the nerve centres reflected in the same way, and with the same direful consequences. In truth, for all practical purposes, the two conditions may be discussed under one head and with the same purpose, that of eradicating the pelvic disease, with the design, or rather with the result, of curing the general disease, in my judgment a result that has been claimed unwarrantably for the cure of certain forms of insanity. If, nevertheless, the theory must be questioned and is unproved facts remain to support, apparently, the abdominal brain and intestinal-pelvic finely-wrought conception of my learned friend, Byron Robinson of Chicago, the *facile princeps* of the pelvic reflex doctrine. The harm that has followed from insisting upon, emphasizing and widely proclaming the doctrine is that men's minds have been unduly turned in this direction. The theory is ridden as a hobby horse with Quixotic zeal and alertness to the detriment of many patients that have been sacrificed upon its altar where presides the scalpel, or other instrument of dread, or of septic conveyance.

---

*Even in the normal state of the economy, "reflex action" is in danger of being dethroned from its exalted position (Loeb).

In a large city recently I attended a clinic for female diseases. A young unmarried girl was exposed before the class, several members of which were required to examine her and make a diagnosis. This performance was completed by the professor in charge. The concensus of opinion was that the patient was suffering from retroversion of the uterus, whereupon the professor declared the condition to be distinctly pathological and the cause of the nervous perturbations, physical weakness and dysmenorrhea from which she suffered. The abnormality was illustrated upon the blackboard, in contrast with the assumed normal position of the uterus. He proposed two operations for the patient's relief: First, curettage—a truly high-sounding scientific term—and second, a choice of any of several operations, extra and intra abdominal, for shortening the ligaments. The history as read said not a word about the miserable conditions in which this poor waif of charity probably lived and had lived from her very birth. What a record of mental and physical misery this history might have disclosed: cruel parents, or no parents, the squalidity and poverty of the average tenement house with its notorious overcrowding and filth, foul air from the absence of sunlight and ventilation, the want of substantial food and clothing; all of these uniting with the strain of eking out a precarious living upon the streets, in the stress of all kinds of weather to wreck utterly a nervous system already made unstable, probably, through hereditary influences. Surely in all this was sufficient cause for arousing at least a suspicion that depressing influences had arrested normal development, not only of the pelvic organs, but the very nerve centers themselves. Why was the class not told that, for this girl, the retroversion might be normal, and that simply because the strands of Mueller had united at a false angle this in all circumstances was not abnormal? Why was not the lesson impressed that the dysmenorrhea, as well as the other symptoms, might be expressions of a general condition of which they only formed a part? Said the experienced and conscientious Joseph Price: "It is wonderful what iron and good hygienic conditions frequently will do in cases that might be considered as requiring operative procedures."

Therefore, this nervous and physical weakness, so often associated with, or caused by abdominal disease in women is not true neurasthenia, or strictly pelvic-reflex—it is so-called. There is a distinction between these conditions and careful effort should be made always to differentiate them before ill-advised operations are undertaken on the quick assumption of cause and effect that would tend to establish their identity contrary to an experienced professional judgment. But the college clinics must be supplied with material for their congested amphitheatres; and now when these institutions, like the ubiquitous streptococcus, are multiplying in the vital stream of the body politic, and if, as Frank Lydston of Chicago predicts, all of us are likely to be professors—that notoriously are averse to operations upon themelves—the material for exemplifying medical teaching must be drawn almost wholly from the female class. When the latter is about extinct Othello's occupation will be gone, and general medicine, that is now suffering, will claim its long-suppressed birthright to restrain, no less than to support, the inordinately ambitious and inconsiderate gynecologist of the future. Even at the present time, assuredly there can be found enough gross conditions of disease in the abdomen of women upon which the aggressive gynecologist can wreak his skill in diagnosis, and his mechanical art in operating.

A short time ago I was summoned to the bedside of a lady living sixty miles from Dallas. Her case was diagnosticated as some obscure abdominal disease, that probably might require an operation for its removal. She was surrounded by anxious relatives and friends, who shared with her a great anxiety as to her condition. Her medical attendant, a very intelligent man, was very apprehensive. Now, note the following clinical history of this patient: She was past fifty years of age, the menses having ceased about six years previously, the menopause being attended by its ordinary phenomena. For several years she had suffered from indigestion and nervous symptoms, these now had reached a climax in her present sickness. She was troubled with cardio-vascular disturbances and pains in the cerebro-spinal nerve centers. There was tympany of the abdomen over which there was much sensitiveness to pressure. She slept little and was troubled with much gastric derangement. Mental and physical effort were distressing. A physical examination showed the tenderness of the abdominal walls to be superficial. Examination *per vaginam* found tenderness in the pelvis, but this was decided to be of a pseudo character, notwithstanding a small retroverted uterus fixed in old pelvic adhesions long since organized, was discovered. For several weeks she had been in constant attendance upon an only son sick with typhoid fever. Worn out by constant watching and solicitude, she had emerged from this trial without any serious breakdown when her son experienced a relapse of his previous condition. This additional weight of care and fear had completely prostrated her, when all the symptoms of a profound neurasthenia showed themselves and treatment for this condition brought manifest and radical improvement. This case briefly narrated is not unique, and in its history combines two causative factors, namely: a persistence of the nerve derangement incident to the menopause, aggravated by the second factor: intense emotions, loss of sleep and appetite, and fatigue consequent upon the conditions to which for a long period she had been exposed. I have observed that the nervous phenomena of the menopause closely resemble, if they are not identical with, those of neurasthenia, and probably it is true that Tilt, in his classical treatise on the change of life, has described the disease that we now call neurasthenia. At all events, this condition is seen frequently at the change of life, when the nerve centers are more readily impressed, or rather depressed, by circumstances real or imaginary, trivial or serious; and in the fore-front of the clinical picture abdominal symptoms of mimetic character may deceive us into a false position that may prove serious to the welfare of the patient, especially should operative interference be attempted.

*A fortiori,* these assertions, as I have above indicated, apply with much significance to the other extreme of life in the female, that of puberty; and with greater emphasis in individuals of both classes predisposed by heredity to unstable nerve centers.

Under the head of "vague pains" that afflict the female neurasthenic must be mentioned those nerve mimicries of pelvic disease, including the ovaries and the peritoneum. In my opinion such pains are not obsessions strictly but realities. Doubtless they are observed in complicated neurasthenia, and I recall an experience of them principally in mixed cases of hysteria and neurasthenia—a most perplexing problem for the diagnostician.

A noteworthy case of this kind was sent to my hospital for operation. Careful examination of the patient startled me with exquisite tenderness in the ovarian areas and throughout that of the entire pelvic peritoneum. Under an anesthetic no exudates whatever were apparent, the pelvic organs being mobile and normal in size and position. Taking this fact in conjunction with the nervous disturbances prevailing elsewhere, and the personal history led me to correct interpretation of the symptoms and possibly to an escape from at least an exploratory laparotomy. The patient was cured by remedies addressed to an irritable nervous system, much appeased also by a "wise neglect" of all local treatment. Such a peritonitis is nervous, and, by Howard Kelly, has been put down in medical terminology as "pseudo-peritonitis" (Herman). It will be found, in the mixed form of the disease, along with the anesthetic areas so minutely described by Lomer and others, as occurring in the abdominal walls over the ovarian region—"ovarie"—in the throat, and at other points in the distribution, chiefly of the superficial nerves. Eye symptoms, also, may be present to strengthen the diagnosis.

One of the most common and intractible symptoms in the neurasthenia of women is mucus (membranous) colitis (?)—Boas *contra*. It is so common in my experience that, invariably, I assume it to be present either in small or in large degree. As a symptom of serious import in neurasthenic cases, it is widely known and merited a full discussion of its diagnosis and treatment by the International Medical Congress several years ago. It may exist separately, or with enteric and gastric derangement, the latter being the ordinary trophic condition ("secretory neurosis") that probably involves the musculature and the glandular surface of the entire alimentary canal.

So obtrusive is this disorder, that it may, more or less, obscure the other symptoms of neurasthenia, with which it is related. It is a veritable *bête-noir* of the physician in the treatment of neurasthenic cases, where it involves the digestive tube to any large extent. Frequently it will be associated with a retro displaced, and, perhaps, a uterus enlarged by sub-involution or metritis with adhesions, the relation of cause and effect being assumed as an indication for operative measures that might entail disastrous results. My experience with neurasthenia in which colitis is present in the several degrees, has been large, but not always satisfactory.

A patient now under treatment affords a typical example of neurasthenia with colitis of maximum intensity. She is thirty years of age, unmarried, of spare habit of body and of a philosophical turn of mind, inhibiting an exceptionally emotional and sympathetic temperament, the legacy of her ancestors. Moreover, this neurotic feature has been intensified for years by pecuniary reverses, and by many deaths of her immediate kin and relatives. During the last twenty years she has suffered nervous breakdowns in the form of neurasthenia, two notable expressions of which have been colitis and dysmenorrhea, but the clinical picture of neurasthenia is complete. An obsession in her case has been that she has uterine or ovarian disease, until finally, I was compelled to make a local examination, only to discover the pelvic organs normal in every respect. She has resigned herself to the certainty that local treatment, with or without an operation, was required, and, assuredly, all the circumstances of the case might have allured the unwary or the enterprising physician into this illogical and perhaps fatal attitude. She is undergoing the rest and seclusion treatment, while the intense colitis is being attacked by the hot, wet pack method, as carried out by Kellogg at the Battle Creek Sanitarium. Of course, the general treatment of neurasthenia is not neglected. Under this regimen, she is gradually, but substantially, improving and will recover from the most severe attack of neurasthenia that she has yet experienced.

On the other hand, it is claimed that there is a toxic organ of neurasthenia (Rockwell) in females, owing to various causes, but notably, to that of obstinate constipation from nervous atony of the muscular walls of the intestines preceding fermentation, and the formation of poison alkaloids from the retention and perverted conversion of food products. If this ever be the pathological beginning of true neurasthenia, I have not recognized it, although I would not deny its occurrence in cases where the indigestion symptoms of neurasthenia are confined apparently to the stomach, constipation resulting. Such a condition may be classed with that of fœcal anemia, first described by Andrew Clark. Indeed, they may be one and the same, the nervous symptoms being caused by malnutrition of the nerve centres in the brain and spinal cord, or of those in the sympathetic ganglia, or of all combined.

In recent years, much study and theorizing has revolved about the close or the remote connection appearing to exist between displacements of the kidney and typical neurasthenia.

This is still a *question vexata* that has been decided, neither by the statistics of operation, nor by those of medical treatment.

"Glendard's disease" may connote all forms of enteroptosis. Some claim this to be the rule. Operations to fix the displaced kidney in these cases are of doubtful value (Goodhart) and, in some cases, as I have seen, positively harmful to the nervous system. The zeal of operators may influence their statistics as has been shown in a recent study of such statistics by Fitz of Boston, who delares that, so far as regards permanent, beneficial result, surgery has not so much to its credit as we proudly claim for it. Statistics have been said to be the "greatest of all lies;" they may stand as hieroglyphics for facts that may have never existed.

Understand me not as claiming that suturing the displaced kidney to its normal position is never a beneficent procedure. It is the fierce onslanght upon the organ by the mechanical surgeon, without matured consideration of all the pathological questions involved, that invites my scepticism, justified by facts that have come recently to my knowledge. The pioneers of medical and surgical practice are not always prejudged unwisely, or with jealousy. The pronouncement by Koch: *"Ich habe est gefunden,"* may have led not to disaster in medical therapeutics, although it might have been otherwise; but during the present cyclonic disturbances in the surgical department of our science, it behooves the thoughtful surgeon to take shelter within the walls of that protection afforded by the fundamental principles of sound logic and of research, that may guide his hand and conscience.

In the foregoing desultory remarks, I am aware that I have hardly entered the subject of neurasthenia in women. Within the title of the paper is concealed my real design: to add a mite to the note of warning that is being sounded by the conservative men of the profession against the prevailing indscriminate operations in abdominal cases—operations that rest upon no sound pathological basis. In 1879, the lamented Goodell wrote the following: "We are prone to base far-reaching conclusion on fragmentary evidence, or on indifferent data." Twenty-three years later this dictum is not less opportune; in fact, it should be made more emphatic. "In the yet young and brilliant school of gynecology," there is a mad rush for fame that, it appears, can be secured by the knife only, or else all pathology in the abdominal cavity can only thus be eliminated—an admission discreditable to an advancing profession. The surgical specialist is the hero of the hour, and may be likened to that mythological executioner who forcibly stretched out his victims, or retrenched their pedal extremities, to make them fit the only bed he possessed; he is likely to ignore also the fact that complete recovery from an operation is not proof that this was necessary, or desirable even as a tentative measure. The deductions of a conscientious and well-trained mind find a more solid basis than this doubtful reasoning that, in this era of antiseptic surgery, is made more possible and dangerous. But the demands of society and the profession itself will yet enable general medicine, and diagnosis, and pathology to assert their authority to broaden, restrain and direct us, not in the perplexities of our medical relations alone, but our surgical as well. For centuries past, this has been the goal of professional desire unrealized; that this goal will be reached in the twentieth century, is my belief.

